# Astaxanthin Prevents Mitochondrial Impairment Induced by Isoproterenol in Isolated Rat Heart Mitochondria

**DOI:** 10.3390/antiox9030262

**Published:** 2020-03-23

**Authors:** Olga Krestinina, Yulia Baburina, Roman Krestinin, Irina Odinokova, Irina Fadeeva, Linda Sotnikova

**Affiliations:** Institute of Theoretical and Experimental Biophysics, Russian Academy of Sciences, 142290 Pushchino, Moscow Region, Russia; byul@rambler.ru (Y.B.); rkrestinin@bk.ru (R.K.); odinokova@rambler.ru (I.O.); aurin.fad@gmail.com (I.F.); linda_sotnikova@mail.ru (L.S.)

**Keywords:** mitochondrial respiration, the activity of respiratory chain complexes, ATP synthase activity, oxidative phosphorylation, isoproterenol

## Abstract

Mitochondria are considered to be a power station of the cell. It is known that they play a major role in both normal and pathological heart function. Alterations in mitochondrial bioenergetics are one of the main causes of the origin and progression of heart failure since they have an inhibitory effect on the activity of respiratory complexes in the inner mitochondrial membrane. Astaxanthin (AST) is a xanthophyll carotenoid of mainly marine origin. It has both lipophilic and hydrophilic properties and may prevent mitochondrial dysfunction by permeating the cell membrane and co-localizing within mitochondria. The carotenoid suppresses oxidative stress-induced mitochondrial dysfunction and the development of diseases. In the present study, it was found that the preliminary oral administration of AST upregulated the activity of respiratory chain complexes and ATP synthase and the level of their main subunits, thereby improving the respiration of rat heart mitochondria (RHM) in the heart injured by isoproterenol (ISO). AST decreased the level of cyclophilin D (CyP-D) and increased the level of adenine nucleotide translocase (ANT) in this condition. It was concluded that AST could be considered as a potential mitochondrial-targeted agent in the therapy of pathological conditions associated with oxidative damage and mitochondrial dysfunction. AST, as a dietary supplement, has a potential in the prevention of cardiovascular diseases.

## 1. Introduction

Mitochondria play a key role in the normal functioning of the heart, as well as in the pathogenesis and development of various heart diseases [1]. With increased body’s oxygen demand, e.g., during increased stress or adrenergic stimulation, the heart should be able to rapidly increase the content of ATP. Alterations in mitochondrial bioenergetics are the main cause in the onset and progression of myocardial ischemia [2], resulting in the inhibition of the activity of respiratory complexes and an increased proton leakage from the inner mitochondrial membrane [3]. 

Astaxanthin (AST) is a xanthophyll carotenoid of mainly marine origin with strong antioxidant and anti-inflammatory effects, which have been shown in both experimental and clinical studies. It has both lipophilic and hydrophilic properties [4]. Many red-colored marine animals (e.g., salmons, shrimps) and a few bird species (e.g., flamingo) receive AST orally with the food they eat [5]. AST is considered to be a strong antioxidant and is used as a common antioxidant agent or as a dietary supplement intended for human, animal, and aquaculture consumption. The compound has been implicated in numerous health benefits in humans, including the enhancement of the immune response and reduction in the incidence of cardiovascular diseases and various cancers [6,7,8]. It is common knowledge that AST is capable of maintaining the integrity of mitochondria by preventing oxidative stress [9]. In addition, the carotenoid is able to improve the functional state of rat heart mitochondria (RHM), increasing the respiratory control index (RCI) and the P/O ratio after its direct addition to isolated RHM and in RHM of rats preliminarily treated with AST [10]. Wolf and coauthors proved that AST sustained the mitochondrial function by protecting the mitochondrial redox balance [9]. AST significantly diminishes physiologically occurring oxidative stress and maintains mitochondria in a more reduced state even after stimulation with H_2_O_2_. It also prevents a loss of the membrane potential (ΔΨ_m_) and increases oxygen consumption by mitochondria. AST might prevent mitochondrial dysfunction by permeating the cell membrane and co-localizing within mitochondria [11,12]. AST inhibits oxidative stress in myocardial and SH-SY5Y cells [13,14]. Pongkan et al., showed the effect of AST on mitochondrial dysfunction in ischemic mice. They observed that treatment with AST reduced the production of mitochondrial reactive oxygen species (mtROS), as well as the depolarization and swelling of mitochondria. Mitochondrial dysfunction associated with ischemic myocardial injury is facilitated by AST [15].

The researchers of some laboratories have shown that AST decreases the formation of oxidative damage products, increases antioxidant enzyme activity, suppresses inflammatory signaling, and reduces lipid peroxidation in the heart [16,17,18,19,20]. Moreover, Suhn Hyung Kim and coauthors observed that *Helicobacter pylori* infection reduced the level of Mn superoxide dismutase 2 (SOD-2) and SOD activity, which might increase the production of mtROS in gastric epithelial adenocarcinoma cells, while AST inhibited the decrease in the activity of mitochondrial SOD and of mitochondrial oxidative stress in infected cells [21].

In various models of heart failure, numerous defects in the electron transport chain (ETC) complexes have been described [22]. The defects in respiratory complexes and ATP synthase may diminish the ATP production and cause the accumulation of reducing equivalents, e.g., NAD(P)H, which suppress substrate oxidation and may lead to mitochondrial dysfunction [23]. Chronic treatment with another antioxidant, melatonin, at a pharmacological dose, has been found to affect the mitochondrial function [24,25,26]. Recently, we showed that the administration of melatonin produced a protective effect in RHM isolated from rats injected with isoproterenol (ISO) [27].

ATP synthase plays a dominant role in maintaining not only the energy state of the cell but also the mitochondrial respiratory function [28]. It is known that sector F_o_ consists of a subunit *c* ring (comprising 12 copies) and one copy of each of subunits *α*, *b*, *d*, and *h* (F6) [28]. The subunit *c* (ATP5G) is a mitochondrial N, N-dicyclohexylcarbodiimide (DCCD)-binding proteolipid from the F_o_ sector of F_o_F_1_-ATP synthase [29]. ATP synthase catalyzes the final coupling step of oxidative phosphorylation to supply energy in the form of ATP. The changes at the step can crucially affect the respiration of mitochondria and, consequently, cardiac efficiency.

Cyclophilin D (CyP-D), an adenine nucleotide translocase (ANT)-binding mitochondrial matrix peptidylprolyl *cis-trans* isomerase is a target for cyclosporin A, which mediates the inhibition of mitochondrial permeability transition pore opening [30,31]. CyP-D is associated with complex V (CV) and complex I (CI) [32,33]. Beutner and coauthor suggested that CyP-D regulated the activity of oxidative phosphorylation, presumably altering the activity of the respiratory chain and respirasome assembly and inhibiting the activity of ATP synthase and synthasome assembly of ETC complexes [34]. Earlier, we showed that CyP-D was co-localized with 2′,3′-cyclic nucleotide-3′-phosphodiasterase (CNPase) in rat brain and liver mitochondria (RBM, RLM) [35], which was identified in our laboratory [36]. We observed that CNPase was associated with each complex of ETC, was colocalized with the adenine nucleotide translocator (ANT), voltage-dependent anion channel, CyP-D, and α-tubulin [35], and performed the protective function in RBM and RLM [37,38]. 

Isoproterenol (ISO) is a synthetic, non-selective β-agonist, which has positive chronotropic and inotropic effects, but at high doses, it depletes the energy reserve of cardiomyocytes. ISO induces myocardial stress, which results in the development of infarct-like necrosis. ISO is able to cause oxidative stress, which leads to irreversible damage to the membrane [39].

The aim of the present work was to study the effect of the administration of AST on the structure of the heart tissue, the functional state of RHM, the activity of respiratory complexes, and the levels of the main subunits of ETC complexes in mitochondrial impairment induced by isoproterenol (ISO). In addition, we examined how the administration of AST influenced the level of CyP-D, ANT, SOD-2, and CNPase in RHM.

## 2. Materials and Methods

### 2.1. Animals and Treatment

Sixteen Wistar male rats weighing 240–250 g (age two months) were used. Animals were individually housed in a temperature-controlled room (22 °C) and fed a standard diet with full access to water and food. The experiments were carried out according to the Regulations for Studies with Experimental Animals (Decree of the Russian Ministry of Health of 12 August 1997, No. 755). The protocol was approved by the Commission on Biological Safety and Ethics at the Institute of Theoretical and Experimental Biophysics, Russian Academy of Sciences (March 2019, protocol N18/2019). For the experiments, rats were divided into four groups (four rats in each group); thus, four independent replicates were done for each group. The first and third groups of rats served as a control, and the animals of groups 2 and 4 were given AST (Natural, China) every day for 2 weeks (hereafter referred to as AST groups 2 and 4). A weighed quantity of 5% AST (150 mg/kg) [10] was dissolved in olive oil and administered orally using plastic feeding tubes, 15 ga × 78 mm (Instech, Plymouth Meeting, Plymouth, PA, USA). The animals of the control groups received an equal amount of olive oil as a vehicle. After two weeks, the rats of groups 3 and 4 were injected with ISO dissolved in physiological saline (85 mg/kg of body weight) twice at an interval of 24 h to induce mitochondrial disorders [27,40], and the rats of groups 1 and 2 were injected with saline. The next day after the 2nd injection of ISO or saline, RHM was isolated from the hearts of rats in each group.

### 2.2. Histological Analysis

To prevent autolysis, immediately after the withdrawal of the heart (within 10 min after decapitation), the fragments of the left ventricle of the heart were washed for 30 s with a cold (14 °C) isotonic solution and fixed for 24 h in neutral buffered formalin (NBF) at the tissue volume–fixator volume ratio 1:30. After the termination of fixation, the fragments of ventricles were washed with distilled water (3 × 3 min) to remove excessive phosphates and placed for no less than 12 h in medium Optimum Cutting Temperature (O.C.T.) Compound Tissue Tek (Sakura, Tokyo, Japan). Cross slices of heart ventricles of rats (9 μm) were prepared by cryosectioning (Shandon CRYOTOME 620E, Thermo Fisher Sci., Waltham, MA, USA). The staining of samples was performed by a conventional method using H&E (Mayer’s Hematoxylin and Eosin Y) and Masson’s trichrome [41]. For the analysis, analogous regions of the medial part of the left ventricle wall of all samples having a typical structure and orientation of muscle fibers were used. The micrographs of stained histological samples were obtained on a Nikon Eclipse Ti-E microscope station (Nikon, Tokyo, Japan) and processed using the software NIS Elements AR4.13.05 (Build 933).

### 2.3. Isolation of Rat Heart Mitochondria

Mitochondria were isolated from the whole hearts by a standard technique [27]. Each heart was chopped, freed of blood vessels, and destroyed with a glass homogenizer in a tenfold volume of a medium containing 75 mM sucrose, 10 mM Tris-HCl (pH 7.4), 225 mM mannitol, 0.5 mM EDTA, 0.5 mM EGTA, and 0.1% BSA. The homogenate was centrifuged at 1000× *g* for 10 min, and the pellet was removed. Mitochondria contained in the supernatant were sedimented at 8500× *g* for 10 min. Then, the mitochondrial pellet was washed with the isolation medium without EDTA and BSA (8500× *g*, 10 min) and suspended in the same medium. All procedures were performed at 4 °C. The protein content in mitochondria was determined using the Bradford assay. The protein concentration in an RHM suspension was 30–35 mg/mL. 

### 2.4. Evaluation of Mitochondrial Respiratory Functions

Mitochondria (1 mg protein/mL) were incubated at 25 °C in a medium containing 125 mM KCl, 10 mM Tris (pH 7.4), and 2 mM K_2_HPO_4_. In the experiments, glutamate (5 mM) and malate (5 mM) were used as respiratory substrates. The respiratory control index (RCI) was measured in a closed chamber after the addition of 150 μM ADP and 1.5 μM oligomycin to RHM. The oxygen consumption rates (V_st.2_, V_st.3_, and V_st.4_; ng-atom O min^−1^ mg^−1^ of protein) were evaluated.

### 2.5. Blue Native Electrophoresis (BNE) and Measurement of the Activity of Electron Transport Chain Complexes and ATP Synthase 

BNE was performed as described [35]. The buffer containing 0.75 M aminocapronic acid, 50 mM Bis-Tris/HCl, pH 7, and 10% dodecyl maltoside was added to intact RHM, and the suspension was kept on ice for 20 min. After 10-min centrifugation at 10,000 × g, the supernatant was supplemented with 5% Serva Blue G dissolved in 1 M aminocapronic acid. Samples were applied onto 3–13% gradient gel, 70 µg of the sample per lane. An HMW Calibration Kit for Native Electrophoresis (Sigma-Aldrich, St. Louis, MO, USA) was used as molecular markers.

For the measurement of the in-gel activity of complexes, electrophoresis was done on ice. After the BNE, the in-gel activity of complexes I, II, IV, and V was determined as described [42]. Mitochondrial samples were separated by one-dimensional BNE (1D BNE), and the gel was stained for ~10–30 min for the detection of activity of CI by buffer containing 100 mM Tris-HCl, pH 7.4, 0.14 mM NADH, 1 mg/mL NBT (nitro blue tetrazolium chloride). For the detection of complex IV (CIV) activity, the gel was stained for 1 h by a buffer containing 10 mM KH_2_PO_4_ (pH 7.4), 1 mg/mL (3,3′-diaminobenzidine) (DAB), and 0.2 mg of cytochrome *c*. The complex II (CII) activity was determined by staining with a buffer containing 50 mM KH_2_PO_4_ (pH 7.4), 84 mM succinate, 0.2 mM phenazine methosulfate (PMS), and 2 mg/mL NBT. For the detection of CV activity, the gel was stained for 16 h with a buffer containing 10 mM ATP, 35 mM Tris (HCL), 270 mM glycine, 14 mM MgSO_4_, 0.2% Pb(NO_3_)_2_. After incubation in the appropriate substrates, the reactions were stopped with 10% acetic acid, and gels were washed with water and scanned. Two gels simultaneously were run, one gel was used for in-gel activity determination, and another for immunoblotting. The bands of the separated complexes were cut out after electrophoresis and applied in 12.5% SDS-PAGE followed by immunoblotting. Precision Plus Pre-stained Standards markers from Bio-Rad Laboratories (Hercules, CA, USA) were used. 

### 2.6. Preparation of Samples, Electrophoresis, and Immunoblotting of Mitochondrial Proteins

Samples for determining the protein levels were prepared by placing the aliquots of isolated intact RHM from each group in Eppendorf tubes and solubilizing them in Laemmli buffer (Bio-Rad, Hercules, CA, USA). After heating for 3 min to 95 °C, the samples containing 20 μg of mitochondrial protein were applied onto each line and subjected to electrophoresis and then to Western blot analysis. The polyclonal anti-ANT antibody (1:1000), the monoclonal anti-CyP-D antibody (1:1000), the monoclonal anti-ATPG1/G2/G3 antibody (subunit *c*) (1:1000), anti-ATPF1 (subunit *b*), and the polyclonal anti-SOD-2 antibody were from Abcam (Cambridge, UK); the monoclonal anti-CNPase antibody was obtained as described and used at the 1:10,000 dilution [43]. Alterations in the level of subunits of ETC complexes were determined using a Total Oxphos Rodent WB Antibody Cocktail (Abcam, Cambridge, UK). The Oxphos Antibody Cocktail consists of complex V alpha subunit (CV-ATP5A-55 kDa), complex III core protein 2 (Cytochrome b-c1 complex subunit 2, CIII-UQCRC2-48 kDa), complex IV subunit I (mitochondrially encoded cytochrome c oxidase I, CIV-MTCO1-40 kDa), complex II subunit 30 (Succinate dehydrogenase [ubiquinone] iron-sulfur subunit, CII-SDHB-30 kDa), and complex I subunit NDUF8 (NADH dehydrogenase (ubiquinone) 1 beta subcomplex subunit 8, CI-NDUFB8-20 kDa). The monoclonal COX IV antibody (1:1000 dilution; Abcam, Cambridge, UK) and the polyclonal antibody Tom20 (Santa-Cruz, Dallas, TX, USA) were used as a loading control. Immunoreactivity was detected using an appropriate secondary antibody conjugated to horseradish peroxidase (Jackson Immuno Research, West Grove, PA, USA). The blot was detected with ECL (Bio-Rad, Hercules, CA, USA) using the ChemiDoc Touch Imaging System (Bio-Rad, Hercules, CA, USA). Protein bands were quantified by densitometry (Image Lab program, Hercules, CA, USA).

### 2.7. Statistical Analysis

For statistical analysis, relative levels of protein density were expressed as means ± SDs from at least four independent experiments. The statistical significance of the differences between the pairs of mean values was evaluated using the Student-Newman-Keul test (Supplementary Materials, Figure S1 and Table S1). The difference was considered significant at *p* < 0.05.

## 3. Results

### 3.1. Histological Analysis of Cryosections of the Left Ventricle of Rat Heart

**Figure 1:**
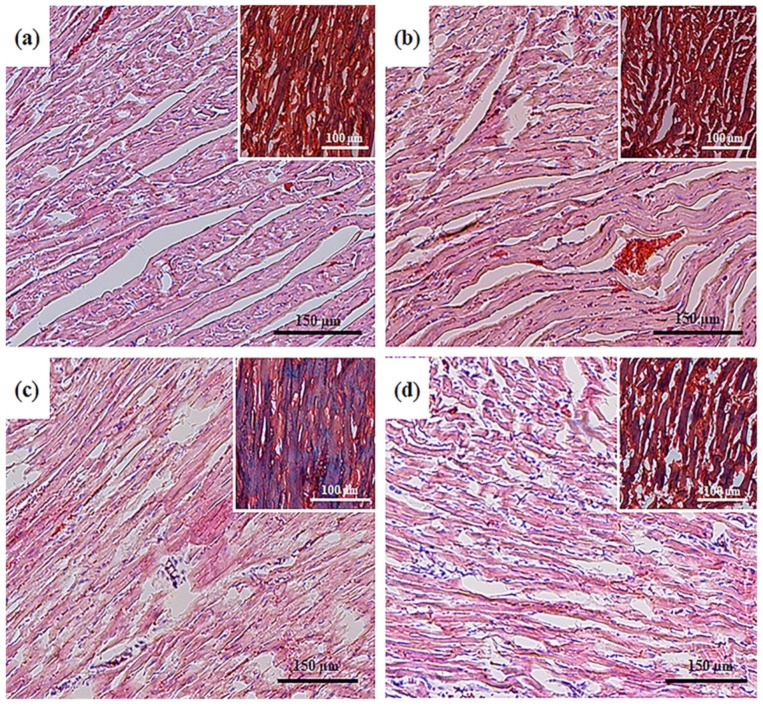
Histological samples of heart ventricles in rats of the control and experimental groups. (**a**) control, group 1; (**b**) chronic astaxanthin (AST) pretreatment, group 2; (**c**) Isoproterenol (ISO) injection, group 3; (**d**) ISO injection and chronic AST pretreatment, group 4. Light microscopy; the main figures show samples stained with hematoxylin and eosin (H&E, cell nuclei are colored blue, erythrocytes are colored red, and muscle tissue is colored pink); internal insets: Masson’s trichrome staining (collagen/fibrosis is colored blue, muscle and other tissues are colored red, cell nuclei are colored brown).

To detect structural changes in heart tissues and a potential cardioprotective effect of AST, we performed a histological analysis of the left ventricle tissue in the following groups of rats: control (group 1); animals exposed to chronic pretreatment with AST (group 2); animals injected with ISO (group 3); animals injected with ISO and then exposed to chronic pretreatment with AST (group 4). Histological analysis showed that the samples of groups 1 and 2 corresponded to standard characteristics of the structure and architectonics of the myocardium of this age group of Wistar rats. In the samples of group 3, degenerative changes in myocardial muscle fibers were observed, which manifested themselves in the intermuscular stroma edema, swelling and fusion of muscle fibers, the emergence of subsegmental contractures, and the development of small foci of necrosis of tissue with lysed cell nuclei (Figure 1c). 

In the case of trichrome staining, in the foci of the most pronounced degenerative changes, the regions of myolysis and interstitial fibrotic alterations that appear in their places were observed, which indicated the initial stages of intensive formation of scar tissue (Figure 1c, inset, blue staining). In samples of group 4, degenerative changes in the myocardium were also observed; however, the processes of fragmentation, deformation, and necrosis of muscle fibers were significantly less pronounced (Figure 1d). No lytic and pycnotic changes in the nuclei of cells of muscle tissue were observed in samples from animals of this group; however, the signs of focal infiltration were observed, indicating the intensive phase of repair inflammation (Figure 1d). Trichrome staining also revealed the swelling of muscle fibers; however, fibrotic alterations were comparatively less pronounced with complete retention of integrity of both muscle fibers and cell nuclei, indicating that there was no myolysis or its initial replacement by fibrotic tissue in these regions (Figure 1d, inset).

### 3.2. Effect of Administration of AST and ISO on Respiratory Activity in Rat Heart Mitochondria 

**Figure 2:**
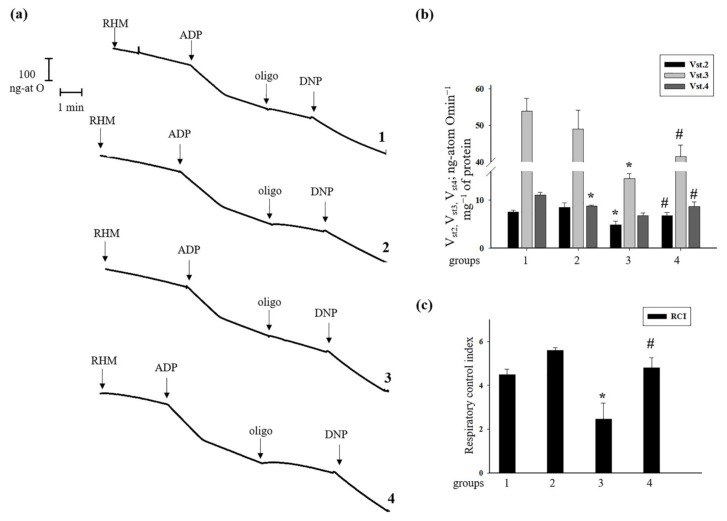
Effects of administration of AST and ISO on respiratory activity in rat heart mitochondria. Rat heart mitochondria (RHM) were incubated in standard medium, as described in Materials and Methods. Arrows show the times at which ADP (adenosine 5‘-diphosphate, 150 µM), oligo (oligomycin, 1.5 µM), and DNP (2,4-dinitrophenol, 30 µM) were added. (**a**) Curves of respiratory activity; (**b**) quantitative analysis of RHM respiration rate in states 2, 3, and 4; (**c**) RCI (respiratory control index) values. The data are presented as the means ± SDs of four independent experiments. * *p* < 0.05 compared with control (group 1). # *p* < 0.05 compared with RHM isolated from ISO-injected rats (group 3).

Further, we measured the oxygen consumption rates in states 2, 3, 4, and the RCI in RHM isolated from rats of each group (Figure 2). Figure 2a shows representative curves of the respiratory activity of RHM. The oxygen consumption rates in different states are given in Figure 2b. It was seen that there were no substantial differences in the oxygen consumption rate in states 2 and 3 in RHM from group 2 of rats that received AST for two weeks compared to group 1. On the contrary, the oxygen consumption rate in states 2 and 3 in RHM of rats injected with ISO decreased by 36 and 73%, respectively, in comparison with RHM from group 1. After the administration of AST followed by ISO injection, the oxygen consumption rate in states 2 and 3 in RHM increased by 40% and 2.8 times, respectively, compared to RHM of group 3. The oxygen consumption rate in state 4 in RHM from rats of group 2 slowed down by 20% compared with group 1. The oxygen consumption rate in state 4 in RHM isolated from group 3 decreased by 50%, respectively (RHM from group 3 vs. group 1). Pretreatment with AST before ISO injection did not change the oxygen consumption rate in state 4 in RHM from group 4. Figure 1c gives the RCI for RHM from each group of animals. The RCI of RHM from group 2 increased by 24% and was approximately 5.6, whereas the RCI of RHM from group 1 was 4.5. The RCI from group 3 decreased 1.8 times compared to the RCI for group 1 and was 2.5. The administration of AST, in combination with ISO, increased the RCI two times relative to the RCI of group 3 and was 4.8. The pretreatment with AST improved the functional state of RHM. 

### 3.3. Effect of AST and ISO on the Level of Enzymes in the Electron Transport Chain in Intact Rat Heart Mitochondria

**Figure 3:**
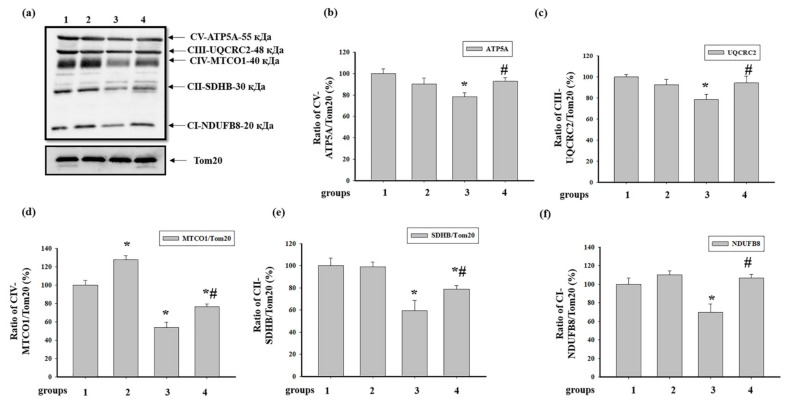
Effect of the administration of AST and ISO on the level of subunits of mitochondrial respiratory chain complexes in rat heart mitochondria. Protein samples were extracted and subjected to Western blotting. Changes in mitochondrial complexes were detected using a Total OXPHOS Rodent WB Antibody Cocktail. The immunodetection of Tom20 was used as a loading control. (**a**) Immunostaining with the OXPHOS antibody cocktail and Tom20; (**b**–**f**) Quantification of immunostaining by computer-assisted densitometry (subunit α of complex V (CV-ATP5A-55 kDa), cytochrome b-c1 complex subunit 2 of complex III (CIII-UQCRC2-48 kDa), mitochondrially encoded cytochrome *c* oxidase I subunit of complex IV (CIV-MTCO1-40 kDa), succinate dehydrogenase [ubiquinone] iron-sulfur subunit of complex II (CII-SDHB-30 kDa), and dehydrogenase [ubiquinone] 1 β subcomplex subunit 8 of complex I (CI-NDUFB820 kDa), respectively). Bar graphs represent the levels of appropriate complexes (I–V); the data are presented as the means ± SD. * *p* < 0.05 significant difference in the protein level in comparison with the control (group 1). # *p* < 0.05 compared to RHM isolated from ISO-injected rats (group 3). The statistical significance of the differences between the pairs of mean values was evaluated using the Student-Newman-Keul test (Supplementary Materials, p. 1).

We analyzed changes in the content of the basic subunits of ETC enzyme complexes in intact RHM isolated from each group of rats (Figure 3). Figure 3a shows a Western blot stained with Oxphos antibodies to the basic subunits of the ETC complexes. Tom20 was used as a loading control. Figure 3b–f demonstrate the data on immunostaining obtained by computer-assisted densitometry and represent the ratios of proteins to Tom20. We observed that the injection of ISO decreased the levels of subunits of CV and CIII approximately by 20% (RHM from group 3 vs. group 1, Figure 2b), while AST abolished the effect of ISO and increased the level of these subunits up to control levels (RHM from group 4 vs. group 3, Figure 3b,c). The level of the NADH-dehydrogenase subunit B8 of CI diminished by 30% in RHM from rats injected with ISO compared to the control (RHM from group 3 vs. group 1, Figure 3f). AST eliminated the effect of ISO and increased the level of the subunit of CI by 40% (RHM from group 4 vs. group 3). We did not notice any changes in the levels of CV, CIII, and CI subunits in RHM from the rats of the AST and the AST+ISO groups in comparison with RHM from the control group (Figure 3b,c,f). The AST alone increased the content of the cytochrome *c* oxidase 1 of complex IV by 28% (RHM from group 2 vs. group 1), while ISO diminished the level of the subunit by 50% relative to that of RHM from the control group (Figure 3d) (RHM from group 3 vs. group 1). The combined action of AST and ISO led to an increase in the level of the CIV subunit by 20% relative to RHM from rats injected with ISO (RHM from group 4 vs. group 3) but did not reach the control level. In RHM from rats treated with ISO and ISO in combination with AST, the level of succinate dehydrogenase B of CII decreased by 40 and 20%, respectively (RHM from groups 3 and 4 vs. group 1, Figure 3e). However, ISO, in combination with AST, upregulated the level of succinate dehydrogenase B of CII by 20% compared to RHM from ISO-injected rats (RHM from group 4 vs. group 3). 

### 3.4. Effect of Administration of AST and ISO on the Level of Proteins Associated with the Complexes of the Electron Transport Chain in intact Rat Heart Mitochondria 

**Figure 4:**
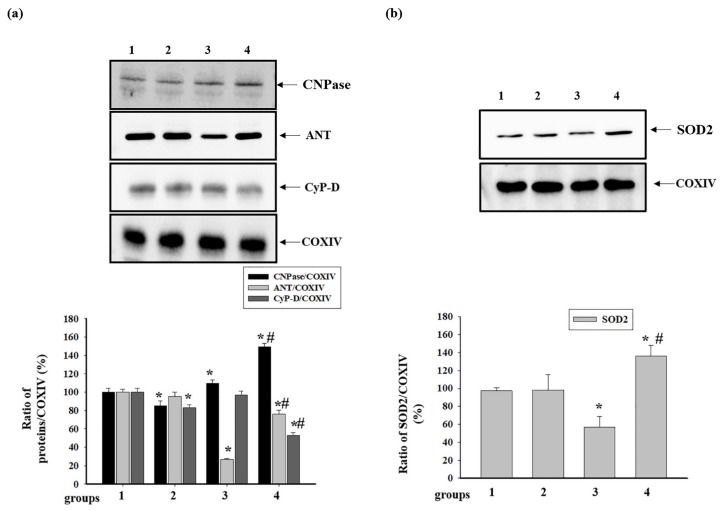
Effect of the administration of AST and ISO on the content of mitochondrial proteins: cyclophilin D (CyP-D), adenine nucleotide translocator (ANT),2′,3′-cyclic nucleotide-3′-phosphodiasterase (CNPase), and superoxide dismutase 2 (SOD-2). (**a**) The upper part: Western blots stained with the corresponding antibodies (CNPase, ANT, and CyP-D); the lower part: a diagram quantitatively reflecting changes in protein content in absolute units normalized to COX IV. (**b**) The upper part: Western blots stained with the antibody to SOD-2; the lower part: a diagram quantitatively reflecting changes in the content of SOD-2 in absolute units normalized to COX IV. The data are presented as the means ± SDs of four independent experiments. * *p* < 0.05 significant difference in protein level compared with the control (group 1), # *p* < 0.05 compared to RHM from ISO-injected rats (group 3). The statistical significance of the differences between the pairs of mean values was evaluated using the Student-Newman-Keul test (Supplementary Materials, p. 2).

Since ANT, CyP-D, and CNPase are associated with some complexes of the ETC, we examined the level of these proteins in intact RHM isolated from the rats of each group (Figure 4). Figure 4 (the upper part) shows Western blots of the proteins in RHM. The results of a quantitative analysis of protein levels are shown in the lower part of Figure 4. Protein bands were quantified after normalization to COXIV. The levels of CNPase and CyP-D decreased by 15% and 17%, respectively, in RHM from rats pretreated with AST, while the level of ANT did not change (RHM from group 2 vs. group 1). Treatment with ISO led to an increase in the level of CNPase by 10% and a decrease in the level of ANT by 70%, whereas the level of CyP-D did not change (RHM from group 3 vs. group 1). AST, in combination with ISO, increased the level of CNPase by 50% compared to the control (RHM from group 4 vs. group 1) and by 28% relative to the effect of ISO alone (RHM from group 4 vs. group 3). Under these conditions, the levels of ANT and CyP-D decreased by 24 and 47%, respectively, compared with the control (RHM from group 4 vs. group 1). However, AST used in combination with ISO increased the level of ANT three times and diminished the level of CyP-D by 46% compared to the effect of ISO alone (RHM from group 4 vs. group 3). Moreover, we investigated changes in the level of SOD-2 in RHM from animals of each group (Figure 3b). Treatment with ISO resulted in a decrease in the level of SOD-2 by 43% (RHM from group 3 vs. group 1), whereas AST increased the content of SOD-2 by 35% compared with the control (RHM from group 4 vs. group 1) and two times relative to RHM of animals with ISO injections (RHM from group 4 vs. group 3). 

### 3.5. Effect of Administration of AST and ISO on the Activity of Respiratory Chain Complexes and the Level of Proteins Associated with Complexes in Rat Heart Mitochondria 

**Figure 5:**
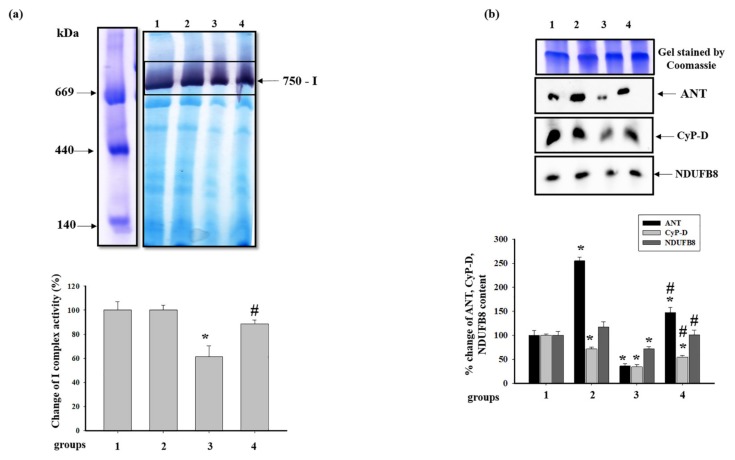
Effect of AST and ISO on the activity of complex I (CI) in rat heart mitochondria. (**a**) The upper part: freshly prepared rat mitochondria isolated from each group were solubilized in a buffer for blue native electrophoresis (BNE) samples (Materials and Methods section). Mitochondrial samples were separated by 1D BNE, and the gel was stained for the detection of the activity of CI by a buffer containing 100 mM Tris-HCl, pH 7.4, 0.14 mM NADH, 1 mg/mL NBT (nitro blue tetrazolium) for ~10–30 min; the lower part, a diagram quantitatively reflecting changes in the activity of CI; (**b**) the upper part: excised bands with enzymatic activity were subjected to 2D SDS-PAGE and blotted on nitrocellulose for immunodetection using the polyclonal antibody against ANT1 and monoclonal antibodies against CyP-D and NDUFB8 (NADH dehydrogenase [ubiquinone] 1 beta subcomplex subunit 8 of CI); the lower part: a diagram quantitatively reflecting changes in protein levels. The data are presented as the means ± SDs of four independent experiments. * *p* < 0.05—significant difference in the protein level compared with the control (group 1), # *p* < 0.05 compared to RHM from ISO-injected rats (group 3). The statistical significance of the differences between the pairs of mean values was evaluated using the Student-Newman-Keul test (Supplementary Materials, p. 3).

Then, we examined how AST and ISO affect the activity of the complexes of the respiratory chain and the levels of proteins associated with the ETC in RHM. The activity of respiratory chain complexes was measured, as described in Materials and Methods. Figure 5a shows changes in the activity of the NADH-ubiquinone oxidoreductase, complex I (CI), after BNE. It was seen that pretreatment with AST did not differ the activity of CI (RHM from group 2 vs. group 1), while ISO diminished the activity of CI by 40% (RHM from group 3 vs. group 1). The combined action of AST and ISO led to an increase in the CI activity in RHM by 27% (RHM from group 4 vs. 3). The difference in CI activities in RHM between groups 1 and 4 was not observed. Figure 5b represents changes in the level of proteins associated with CI, such as ANT, CyP-D, and the NADH-dehydrogenase subunit B8 (NDUFB8) of CI. In RHM from group 2, ANT increased approximately 2.5 times compared to control (RHM from group 2 vs. group 1). ISO diminished the level of ANT by 75%, while ISO in combination with AST upregulated the ANT level in RHM by 47% relative to the control (RHM from group 4 vs. group 1) and four times compared with RHM from ISO-injected animals (RHM from group 4 vs. group 3). The CyP-D content decreased by 28.5 and 65.7% in RHM from groups 2 and 3, respectively, whereas the level of CyP-D in RHM from group 4 diminished by 55% in comparison with that in RHM from the control group but increased by 30% compared to RHM from group 3. We did not notice any changes in the levels of NDUFB8 in RHM from animals of groups 2 and 4 in comparison with RHM from group 1. On the contrary, treatment with ISO diminished the NDUFB8 level by 28% (RHM from group 3 vs. group 1). ISO, in combination with AST, upregulated the NDUFB8 level in RHM by 30% compared with RHM from rats injected with ISO (RHM from group 4 vs. group 3). AST abolished the harmful effect of ISO in RHM.

**Figure 6:**
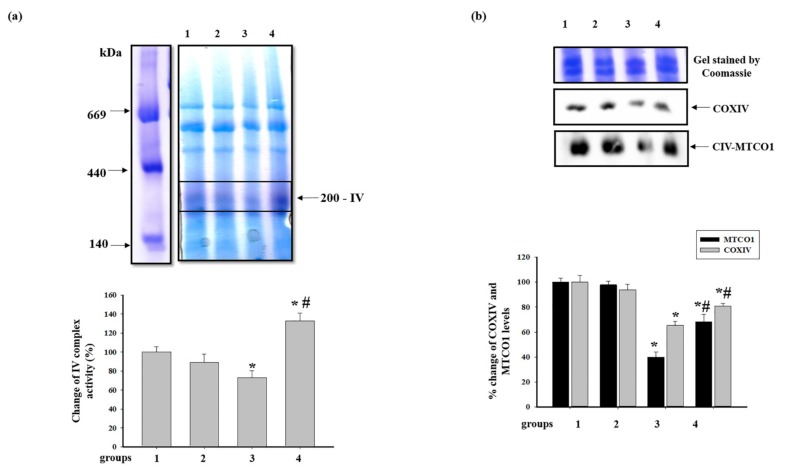
Effect of AST and ISO on the activity of complex IV (CIV) in rat heart mitochondria. (**a**) The upper part: freshly prepared rat mitochondria isolated from each group were solubilized in a buffer for BNE samples (Materials and Methods Section). Mitochondrial samples were separated by 1D BNE, and the gel was stained for the detection of the activity of CIV by a buffer containing 10 mM KH2PO4 (pH 7.4), 1 mg/mL DAB (3,3′-Diaminobenzidine), and 0.2 mg of cytochrome c for 1 h; the lower part: a diagram quantitatively reflecting changes in the activity of CIV; (b) the upper part: the excised bands with enzymatic activity were subjected to 2D SDS-PAGE and blotted onto nitrocellulose for immunodetection using the monoclonal antibodies against COXIV and MTCO1; the lower part: a diagram quantitatively reflecting changes in the protein levels. The data are presented as the means ± SDs of four independent experiments. * *p* < 0.05—significant difference in the protein level compared with the control (group 1), # *p* < 0.05 compared to RHM from ISO-injected rats (group 3). The statistical significance of the differences between the pairs of mean values was evaluated using the Student-Newman-Keul test (Supplementary Materials, p. 4).

The alterations in the activity of cytochrome *c* oxidase, complex IV (CIV), and the level of proteins of CIV are shown in Figure 6. Figure 6a shows changes in the activity of the CIV after BNE. It was seen that pretreatment with AST did not change the activity of CIV (group 2 vs. group 1), while ISO diminished the activity of CIV in RHM by 27% (group 3 vs. group 1).

The combined action of AST and ISO led to an increase in the CIV activity by 90% relative to RHM from rats injected with ISO (RHM from group 4 vs. group 3) and by 30% compared to the control group (RHM from group 4 vs. group 1). Figure 5b represents changes in the level of subunits of CIV, such as cytochrome *c* oxidase 1 (MTCO1) and IV (COXIV). The administration of AST did not change the contents of both proteins (RHM from group 2 vs. group 1). The levels of both proteins in RHM from ISO-injected rats decreased by 60 and 35%, respectively (RHM from group 3 vs. group 1). After combined administration of AST and ISO, the levels of the proteins were by 32 and 20% lower compared with the control (RHM from group 4 vs. group 1); however, compared with RHM from ISO-injected rats, they increased by 72 and 15%, respectively (RHM from group 4 vs. group 3). AST abolished the effect of ISO, increasing the levels of both proteins. 

**Figure 7:**
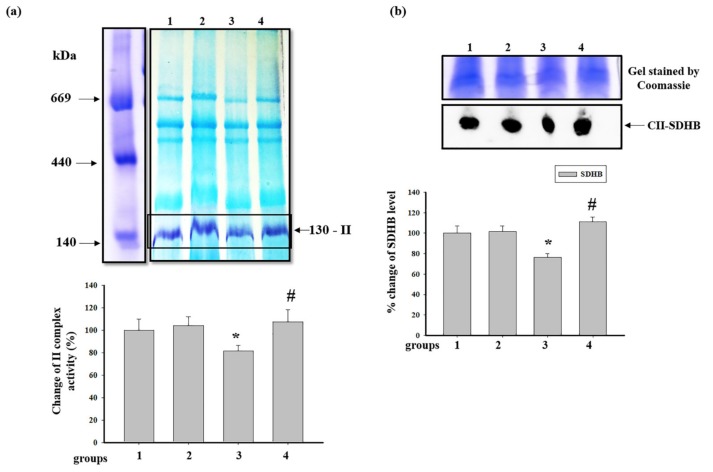
Effect of AST and ISO on the activity of complex II (CII) in rat heart mitochondria. (**a**) The upper part: freshly prepared rat mitochondria isolated from each group were solubilized in a buffer for BNE samples (Material and Methods section). Mitochondrial samples were separated by 1D BNE, and the gel was stained for the detection of the activity of CII by a buffer containing 50 mM KH_2_PO_4_ (pH 7.4), 84 mM succinate, 0.2 mM PMS (phenazine methosulfate), and 2 mg/mL NBT; the lower part: a diagram quantitatively reflecting changes in the activity of CIV; (**b**) the upper part: the excised bands with enzymatic activity were subjected to 2D SDS-PAGE and blotted onto nitrocellulose for immunodetection using the monoclonal antibody against SDHB (succinate dehydrogenase [ubiquinone] iron-sulfur subunit of CII); the lower part: a diagram quantitatively reflecting changes in the protein level. The data are presented as the means ± SDs of four independent experiments. * *p* < 0.05—significant difference in the protein level compared with the control (group 1), # *p* < 0.05 compared to RHM from ISO-injected rats (group 3). The statistical significance of the differences between the pairs of mean values was evaluated using the Student-Newman-Keul test (Supplementary Materials, p. 5).

Then, we measured the activity of succinate-ubiquinone oxidoreductase, complex II (CII) in RHM from each group (Figure 7). Figure 7a shows changes in the activity of CII after BNE. According to the results, no substantial changes were observed in RHM from the animals of group 2 in comparison with RHM from the control group. However, after the injection of ISO, the activity of CII in RHM diminished by 20% compared to RHM from the control group (RHM from group 3 vs. group 1). ISO, in combination with AST, did not change the activity of CII compared to RHM from the control group (RHM from group 4 vs. group 1) and upregulated the activity by 30% relative to RHM from ISO-injected rats (RHM from group 4 vs. group 3). After BNE, the level of succinate dehydrogenase B (SDHB) in RHM from each group was examined (Figure 7b). It was found that the SDHB content did not change in RHM from rats pretreated with AST, while ISO decreased the level of the protein by 25% compared to RHM from the control group (RHM from group 3 vs. group 1). The AST administered in combination with ISO increased the SDHB level by 45% compared to RHM from ISO-injected rats (RHM from group 4 vs. group 3) and affected its level in RHM from the control group (RHM from group 4 vs. group 1).

**Figure 8:**
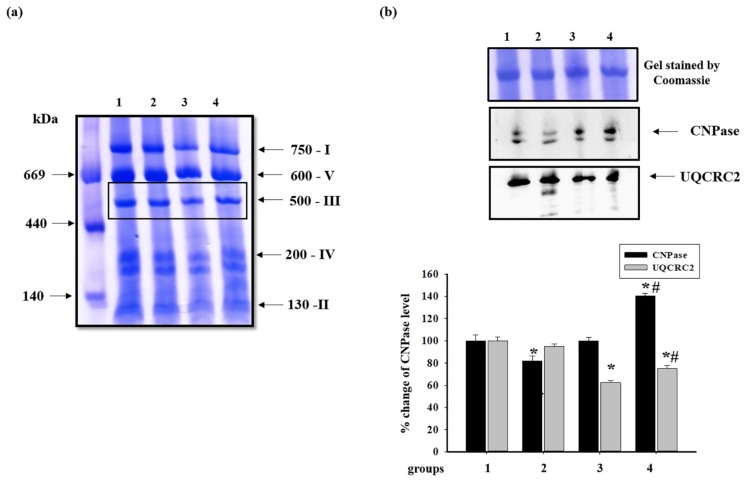
Effect of AST and ISO on the level of proteins associated with complex III (CIII) in rat heart mitochondria. (**a**) Freshly prepared rat mitochondria isolated from each group were solubilized in the buffer for BNE samples (Materials and Methods Section). Mitochondrial samples were separated by 1D BNE, and excised bands were subjected to 2D SDS-PAGE; (**b**) the upper part: Western blot stained with monoclonal antibodies against UQCRC2 (cytochrome b-c1 complex subunit 2) and CNPase; the lower part: a diagram quantitatively reflecting changes in protein levels. The data are presented as the means ± SDs of four independent experiments. * *p* < 0.05—a significant difference in the protein level compared with the control (group 1), # *p* < 0.05 compared to RHM from rats injected with ISO (group 3). The statistical significance of the differences between the pairs of mean values was evaluated using the Student-Newman-Keul test (Supplementary Materials, p. 6).

Then, changes in the content of cytochrome bc1 complex subunit 2 (UQCRC2) of the ubiquinol-cytochrome *c* oxidoreductase, complex III (CIII), was examined (Figure 8). Figure 8b shows changes in the level of proteins after BNE (Figure 8a). It was seen that the level of UQCRC2 in RHM from ISO-treated rats decreased by 40% compared to RHM from the animals of the control group (RHM from group 3 vs. group 1) and did not change in RHM from rats pretreated with AST (RHM from group 2 vs. group 1). AST, in combination with ISO, decreased the level of UQCRC2 by 25% compared to RHM from the control group (RHM from group 4 vs. group 1) and increased it by 17% relative to RHM from ISO-treated rats (RHM from group 4 vs. group 3). Recently, we revealed that CNPase was associated with all complexes of the ETC in RBM [35]. In the present study, we found that CNPase was associated only with complex III and examined changes in its level in RHM from each group. It was shown that the level of CNPase in RHM from rats pretreated with AST decreased by 17% (RHM from group 2 vs. group 1). ISO alone did not change the CNPase level in RHM compared to the control (RHM from group 3 vs. group 1). ISO, in combination with AST, increased the CNPase content in RHM by 40% (RHM from group 4 vs. groups 3 and 1). 

**Figure 9:**
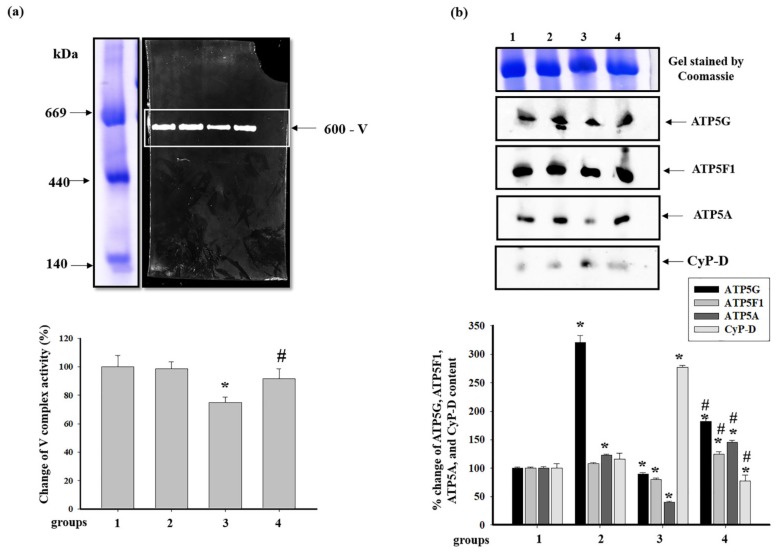
Effect of AST and ISO on the activity of ATP synthase in rat heart mitochondria. (**a**) The upper part: freshly prepared rat mitochondria isolated from each group were solubilized in the buffer for BNE samples (Materials and Methods section). Mitochondrial samples were separated by 1D BNE, and the gel was stained for 16 h for the detection of complex V (CV) activity by a buffer containing 10 mM ATP, 35 mM Tris (HCL), 270 mM glycine, 14 mM MgSO_4_, and 0.2% Pb(NO_3_)_2_; the lower part: a diagram quantitatively reflecting changes in CV activity; (**b**) the upper part: excised bands with enzymatic activity were subjected to 2D SDS-PAGE and blotted onto nitrocellulose for immunodetection using monoclonal antibodies against subunits α (ATP5A), c (ATP5G), b (ATP5F1), and CyP-D; the lower part: a diagram quantitatively reflecting changes in the protein levels. The data are presented as the means ± SDs of four independent experiments. * *p* < 0.05—significant difference in the protein level compared with the control (group 1), # *p* < 0.05 compared to RHM from ISO-injected rats (group 3). The statistical significance of the differences between the pairs of mean values was evaluated using the Student-Newman-Keul test (Supplementary Materials, p. 7).

At the next step, we examined the activity of ATP synthase, complex V (CV) of the ETC. Alterations in the activity of CV and the level of CV subunits and CyP-D associated with CV are shown in Figure 9. It was seen that pretreatment with AST did not change the activity of CV in RHM (RHM from group 2 vs. group 1), while the injection of ISO diminished it by 25% (RHM from group 3 vs. group 1). AST abolished the effect of ISO, and the activity of CV increased by 22% (Figure 9a) (RHM from group 4 vs. group 3). 

The changes in the levels of subunits *c* (ATP5G), *b* (ATP5F1), *α* (ATP5A), and of CyP-D in RHM are shown in Figure 9b. The level of subunits *c* and *α* in RHM from group 2 increased 3.5 times and by 15% in comparison with RHM from the control group, respectively. However, the levels of subunit *b* and of CyP-D did not change (RHM from group 2 vs. group 1). The injection of ISO led to a decrease in the levels of each subunit by 10, 20, and 60%, respectively, whereas the level of CyP-D increased 2.5 times (RHM from group 3 vs. group 1). Pretreatment with AST increased the levels of subunit *c* two times, that of subunit *b* by 40%, and that of subunit *α* three times compared to RHM from ISO-injected rats (RHM from group 4 vs. group 3). On the contrary, the level of CyP-D diminished 3.5 times under these conditions.

## 4. Discussion

It is generally accepted that mitochondria are the main organelles responsible for ATP production in the cell. Mitochondria are vulnerable to oxidative damage, and structural changes occurring upon oxidative disturbances become more pronounced in pathological states [44]. Alterations in mitochondrial functions often are associated with different diseases. A decrease in oxygen content can affect the mitochondrial function and ATP synthesis, causing a significant reduction in cardiac ATP production during the development of myocardial ischemia [45]. To reduce both oxidative damage and the development of various heart diseases, for example, myocardial infarction, considerable attention has been focused on studies aimed at enhancing the protective response of the organism to oxidative stress using different antioxidants. As known, AST has an inhibitory effect on the oxidative stress-induced mitochondrial dysfunction in myocardial and SH-SY5Y cells [46]. It penetrates through the bilayer membrane, providing protection against oxidative stress and can scavenge ROS and free radicals (superoxide anion, hydrogen peroxide, singlet oxygen, etc.) in both the inner and outer lipid layers of the cellular membrane [47]. AST is able to prevent mitochondrial dysfunction by permeating and co-localizing within mitochondria [11,12]. Recently, we have shown that AST improves the resistance of RHM to Ca^2+^-dependent stress and assumed that this antioxidant could be considered as an effective tool for improving, in general, the functioning of the heart muscle under normal and clinical conditions [10]. Therefore, we suggested that AST is a potential mitochondria-targeted therapeutic agent. In this study, histological analysis of the left ventricular tissue of all groups of rats was performed to detect changes in the structure of heart tissues. The effects of the administration of AST and ISO on the functional state of RHM, the activity of respiratory complexes, and the levels of the main subunits of ETC complexes were examined. Moreover, we investigated how the administration of AST and the injection of ISO influenced the level of CyP-D, ANT, SOD-2, and CNPase in RHM. 

We showed that ISO caused degenerative changes in the muscle fibers of the myocardium, which manifested themselves in the edema of the intermuscular stroma, swelling, and fusion of muscle fibers. The appearance of foci of myolysis and subsegmental contractures, as well as the development of small focal tissue necrosis with lysed cell nuclei in the muscle of the left ventricle of ISO-injected rats, was observed (group 3, Figure 1c). After the administration of AST to rats followed by ISO injection, the fragmentation, deformation, and necrosis of muscle fibers were less pronounced. Lytic or pyknotic changes in cell nuclei, as well as signs of myolysis, were not seen. We observed the swelling of muscle fibers and focal cell infiltration. However, fibrotic changes were less pronounced, while the integrity of both muscle fibers and cell nuclei was retained, indicating the absence of myolysis or its initial replacement with fibrotic tissue in these areas (group 4, Figure 1d).

The effectiveness of mitochondria in promoting oxidative phosphorylation is indicated by the RCI. The injection of ISO led to changes in mitochondrial respiratory rates and a decrease in the RCI (group 3). AST increased the RCI in both mitochondria from rats pretreated with AST (group 2) and mitochondria from rats injected with ISO (group 4) compared to mitochondria from the control group (group 1), thereby improving the functional state of mitochondria (Figure 2). 

The ETC, which consists of transmembrane protein complexes (I–IV) and the freely moving electron transfer carriers—ubiquinone and cytochrome *c*, exists in the inner mitochondrial membrane. The complexes must be assembled into a specifically configured supercomplex to function properly [48,49]. These assembled components, together with CV, become the basis for ATP production during oxidative phosphorylation. It should be noted that defects in respiratory complexes and ATP synthase have an influence on the mitochondrial function. In our experiments, the level of the main subunits of respiratory chain complexes in RHM from ISO-injected rats decreased, suggesting the development of mitochondrial impairment in rats. AST abolished the effect of ISO and upregulated the content of the subunits in intact RHM (Figure 3). Complexes III, IV, and I are involved in electron pumping and the subsequent generation of a directed proton gradient across the inner mitochondrial membrane. In addition, the extent of damage to mitochondrial proteins increases in different pathology, resulting in decreased mitochondrial efficiency and cellular energy production [50]. Heart failure can inhibit the expression of ETC subunits and decrease their activities [1]. We found that ISO decreased the activity of I, IV, and II complexes in RHM, whereas AST eliminated the effect of ISO and restored their activity (Figure 5a, Figure 6a and Figure 7a). It is generally accepted that the main subunits of respiratory chain complexes are considered to have major impacts on mitochondrial efficiency. It should be noted that a decrease in the level of subunits of these complexes can diminish the activity of complexes and the rate of mitochondrial respiration in RHM from ISO-injected rats (Figure 5b, Figure 6b and Figure 7b). The level of the UQCRC2 subunit of CIII was downregulated in RHM after treatment with ISO in both BNE-cut samples and intact RHM (Figure 3c and Figure 8b). AST increased the expression of subunit UQCRC2 in RHM isolated from ISO-injected rats. Interestingly, ATP could modulate the activity of CIV through subunit IV by allosteric interactions [51]. A decrease in the COXIV level could result in a decline in the CIV activity in RHM from ISO-treated rats. The administration of AST abolished the effect of ISO and raised the content of COXIV and, consequently, the activity of CIV in RHM.

Recently, we showed that CNPase in RBM was associated with each complex of ETC and ATP synthase [35] and performed the protective function in RBM and RLM [25,37,38]. In the present work, we found that CNPase was associated only with CIII (Figure 8b), indicating that this bond had tissue specificity. Moreover, we observed that the level of CNPase increased in both intact RHM and BNE-cut samples (Figure 4a and Figure 8b). We supposed that CNPase fulfills a protective function and can be a target for the effect of AST in RHM. 

Antioxidants provide a protection mechanism that provides the destruction of deleterious ROS and inhibits lipid peroxidation. In this case, free-radical-scavenging enzymes, such as catalase, glutathione peroxidase, and superoxide dismutase, promote the recovery of the antioxidant defense system and inhibition of ROS production [52]. In our experiment, a decrease in the amounts of SOD-2 was found in RHM of ISO-injected rats (group 3, Figure 4b). Pretreatment with AST significantly increased the level of SOD-2 in RHM of ISO-injected rats (group 3, Figure 4b). AST provided significant protection of the tissue against oxidative injury.

ATP synthase plays a central role in both maintaining the cellular energy state and the mitochondrial respiratory function [28]. A decrease in ATP synthase activity strongly affects the mitochondrial respiration and hence cardiac performance since mitochondrial energy disturbances are involved in the development of cardiac pathologies [53]. The CV consists of two functional domains: F_o_ and F_1_. The F_o_ complex contains transmembrane subunits that transport protons from the intermembrane space, and F_1_ is a peripheral complex in the matrix, which binds to nucleotides and inorganic phosphate for ATP synthesis [54,55]. It is known that ATP synthase catalyzes the final coupling step of oxidative phosphorylation to supply energy in the form of ATP. Alterations at this stage can crucially influence mitochondrial respiration and, consequently, cardiac performance. It is well established that cardiac contractility is strongly dependent on mitochondria and that a reduction in the content of myocardial ATP is a key feature of heart failure. In the mammalian mitochondrial enzyme, subunit *α* (ATP5A) is a part of the F_1_ sector, and subunits *c* (ATP5G) and *b* (ATP5F1) are the parts of the F_o_ sector of ATP synthase [28]. In our experiments, ISO decreased the activity of CV in RHM, whereas AST abolished the effect of ISO and increased the activity of the complex. In this case, the level of subunits *α*, *c*, and *b* diminished in BNE-cut samples of RHM isolated from ISO-injected rats (Figure 9b), while AST eliminated the effect of ISO and increased the level of each subunit. Pretreatment with AST increased the level of the subunits of respiratory chain complexes, ATP synthase complexes, and RCI, suggesting that it prevented oxidative damage by increasing the mitochondrial efficiency.

CyP-D is a member of the cyclophilin family of peptidyl-prolyl *cis-trans* isomerases, which resides in the mitochondrial matrix. It is believed that CyP-D is associated with CV [32], CI [33], and CNPase [35], supporting that CyP-D might modulate the permeability transition. The interaction of CyP-D with proteins may be important for the regulation of mitochondrial functions [56]. Giorgio and coauthors showed that a decrease in the level of CyP-D increased the activity of ATP synthase, which was by 50% higher in mitochondria with CyP-D knockout compared to CyP-D wild-type mitochondria [32]. We noticed that a decrease in the RCI in RHM isolated from ISO-injected rats and CV activity could lead to an increase in the level of CyP-D associated with CV, while AST increased the RCI in RHM from ISO-injected rats and CV activity; in this case, the CyP-D content decreased (Figure 9b). Moreover, AST diminished the level of total CyP-D in intact RHM isolated from ISO-injected rats (Figure 4a). In addition, the level of CyP-D associated with CI diminished in RHM from ISO-injected rats when CI activity decreased. AST upregulated the CyP-D content in RHM from ISO-injected rats and hence the CI activity (Figure 5b). The AST-elicited decrease in the CyP-D level in RHM of rats injected with ISO could influence the activity of respiratory chain complexes and ATP synthase, improving mitochondrial bioenergetics.

The primary physiological role of ANT is to control the flux of ADP and ATP. ANT can function as a critical factor in the modulation of the oxidative phosphorylation rate through respiratory control, depending on the developmental and metabolic conditions of supply and demand as well as on the tissue [57]. Apparently, in the heart, under conditions of high workload or hypertrophy, the involvement of myocardial ANT in controlling respiration increases [58]. However, ANT can also play a role in cardiac pathology. The hypertrophic heart displays lower ANT protein levels and ADP-dependent respiratory kinetics, which may contribute to myocardial remodeling and heart failure [57]. We observed that ISO decreased the expression of ANT in RHM in both intact RHM and BNE-cut samples, while AST restored the ANT content in both cases (Figure 4b and Figure 5b). In the mature heart, during hard work, an increased level of ANT was found, which presumably caused a greater sensitivity of oxidative phosphorylation to ADP levels [59]. An increase in the ANT level by AST could improve the mitochondrial respiration in RHM of rats injected with ISO. 

It is known that there are two subpopulations of cardiac mitochondria—subsarcolemmal and interfibrillar mitochondria—which exhibit different susceptibility to Ca^2+^-dependent inhibition of oxidative phosphorylation and the opening of the mitochondrial permeability transition pore [60]. Further investigations of the effects of AST and ISO on the functional state of these mitochondrial subpopulations may give insight into the nature and mechanisms of the phenomena observed. 

The data obtained in the present study suggested that AST could be used as a dietary supplement in the prophylaxis of cardiac diseases.

## 5. Conclusions

In summary, degenerative changes induced by ISO in the muscle fibers of the myocardium, which manifested themselves as the edema of the intramuscular stroma, as well as the swelling and fusion of muscle fibers were identified. The appearance of foci of myolysis and subsegmental contractures, as well as the development of small foci of necrosis in the muscle of the left ventricle of rats injected with ISO, was observed. The administration of AST to rats followed by injection of ISO led to a significant attenuation of fragmentation, deformation, and necrosis of muscle fibers. Moreover, the fibrotic alterations, in this case, were less pronounced, and the integrity of both muscle fibers and cell nuclei were not affected, which indicated that myolysis or its initial replacement by fibrosis did not occur. The results of the study suggested that the pretreatment of rats with AST improved the functional state of mitochondria, increasing the RCI and the activity of respiratory chain complexes and ATP synthase in RHM injured by ISO. We showed that oral administration of AST increased the level of the subunits of respiratory chain complexes and ATP synthase in both intact RHM and BNE-cut samples, suggesting that it prevented oxidative damage by increasing the mitochondrial efficiency. CyP-D appeared to regulate mitochondrial oxidative phosphorylation. The administration of AST diminished the CyP-D content and increased the levels of ANT, the subunits of respiratory chain complexes, and ATP synthase subunits in RHM damaged by ISO, indicating an improvement of the functional state of RHM and respiration, which could be a cause of the increased activity of respiratory chain complexes and ATP synthase. The level of CNPase rose by the action of AST in combination with ISO, supposing the protective effect of the protein in RHM. AST administration produced an increase in the level of SOD-2 in RHM of ISO-injected rats, thereby protecting against oxidative injury. Analyzing the foregoing, we could assume that AST prevents oxidative damage by increasing mitochondrial efficiency. AST displayed a protective effect in RHM isolated from ISO-injected rats and could, therefore, be considered as an effective tool for improving the functioning of the heart muscle, in general, under normal and clinical conditions. We concluded that AST could be a potential mitochondrial-targeted agent in the therapy of pathological conditions associated with oxidative damage and mitochondrial dysfunction. For this reason, dietary supplementation with AST has a potential in the prevention of cardiovascular diseases.

## Supplementary Materials

The following are available online at https://www.mdpi.com/2076-3921/9/3/262/s1, Figure S1: The statistical significance of the differences between the pairs of mean values was evaluated using the Student-Newman-Keul test (Figure 3, Figure 4, Figure 5, Figure 6, Figure 7, Figure 8 and Figure 9), Table S1: Numerical values of relative levels of protein density were expressed as means ± SDs from at least four independent experiments (Figure 3, Figure 4, Figure 5, Figure 6, Figure 7, Figure 8 and Figure 9).
