# Supplementary material for: Astaxanthin Prevents Mitochondrial Impairment Induced by Isoproterenol in Isolated Rat Heart Mitochondria
Source: Antioxidants (Basel). 2020 Mar 23;9(3):262. doi: 10.3390/antiox9030262 (PMC7139515; doi:10.3390/antiox9030262)
Supplement: Supplementary file 1 [file antioxidants-09-00262-s001.pdf]

To Fig 3.

	CV			
	group 1	group 2	group 3	group 4
Mean 1	1,3	0,936	0,845	1,261
Mean 2	1,326	1,209	0,793	1,196
Mean 3	1,404	1,157	1,092	1,17
Mean 4	1,339	1,287	1,014	1,313

	CIII			
	group 1	group 2	group 3	group 4
Mean 1	1,2587	1,1229	0,9502	1,234
Mean 2	1,16	1,1229	0,8761	1,0859
Mean 3	1,1476	0,9625	0,8638	1,0612
Mean 4	1,2217	1,0366	0,9995	1,1476

	CIV			
	group 1	group 2	group 3	group 4
Mean 1	1,8905	2,388	0,9751	1,3731
Mean 2	2,0829	2,3084	1,0149	1,592
Mean 3	2,0696	2,4875	1,194	1,4527
Mean 4	2,0895	2,7263	1,194	1,4527

	CII			
	group 1	group 2	group 3	group 4
Mean 1	0,8463	1,001	0,4732	1,001
Mean 2	0,9919	1,0556	0,6643	0,9009
Mean 3	0,9282	1,1375	0,7644	0,9555
Mean 4	0,9464	1,001	0,637	1,001

	CI			
	group 1	group 2	group 3	group 4
Mean 1	0,8463	1,001	0,4732	1,001
Mean 2	0,9919	1,0556	0,6643	0,9009
Mean 3	0,9282	1,1375	0,7644	0,9555
Mean 4	0,9464	1,001	0,637	1,001

CV

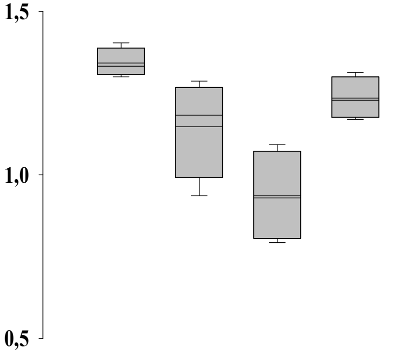

CIII

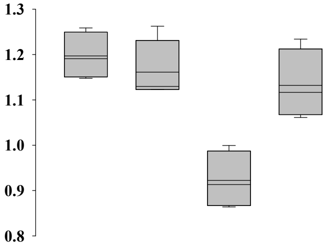

CIV

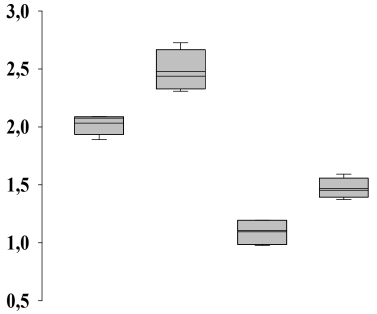

CII

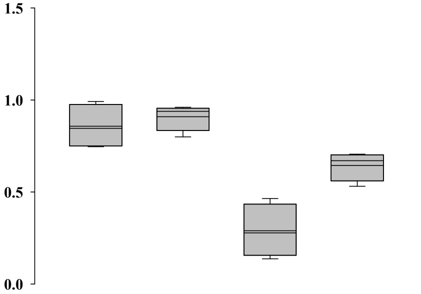

CI

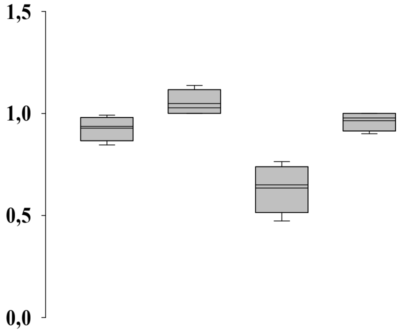

One Way Analysis of Variance All Pairwise Multiple Comparison Procedures (Student-Newman-Keuls Method) :

Comparison	Diff of Means	P<0,050
CV group 1 vs. CV group 3	0,406	Yes
CV group 1 vs. CV group2	0,195	No
CV group 1 vs. CV group 4	0,107	No
CV group 4 vs. CV group 3	0,299	Yes
CV group 4 vs. CV group2	0,0877	Do Not Test
CV group2 vs. CV group 3	0,211	Do Not Test

Comparison	Diff of Means	P<0.050
C III group 1 vs. C III group 3	0.275	Yes
C III group 1 vs. C III group 2	0.21	No
C III group 1 vs. C III group 4	0.139	No
C III group 4 vs. C III group 3	0.136	Yes
C III group 4 vs. C III group 2	0.071	---
C III group 2 vs. C III 3group	0.0648	---

Comparison	Diff of Means	P<0.050
C IV group 1 vs. C IV group 3	0.939	Yes
C IV group 1 vs. C IV group 2	0.248	Yes
C IV group 1 vs. C IV group 4	0.565	Yes
C IV group 4 vs. C IV group 3	0.373	Yes
C IV group 4 vs. C IV group 2	1.01	---
C IV group 2 vs. C IV group 3	1.383	---

Comparison	Diff of Means	P<0.050
C II group 1 vs. C II group 3	0.568	Yes
C II group 1 vs. C II group 2	0.0518	No
C II group 1 vs. C II group 4	0.214	Yes
C II group 4 vs. C II group 3	0.355	Yes
C II group 4 vs. C II group 2	0.265	---
C II group 2 vs. C II group 3	0.62	---

Comparison	Diff of Means	P<0.050
C I group 1 vs. C I group 3	0.293	Yes
C I group 1 vs. C I group 2	0.121	No
C I group 1 vs. C I group 4	0.0364	No
C I group 4 vs. C I group 3	0.33	Yes
C I group 4 vs. C I group 2	0.0842	---
C I group 2 vs. C I group 3	0.414	---

To Fig 4.

CNPase

	group 1	group 2	group 3	group 4
Mean 1	0,9016	0,728	1,212	1,334
Mean 2	0,882	0,4968	0,9936	1,564
Mean 3	0,9199	0,69	1,258	1,288
Mean 4	1,0568	0,7912	1,2992	1,472

ANT

	group 1	group 2	group 3	group 4
Mean 1	1,1776	1,296	0,4352	0,896
Mean 2	1,2672	1,216	0,256	0,96
Mean 3	1,2928	1,1416	0,3584	0,8704
Mean 4	1,3312	1,2544	0,4096	0,8704

Cyc D

	group 1	group 2	group 3	group 4
Mean 1	0,7566	0,585	0,7371	0,4524
Mean 2	0,7176	0,6084	0,7098	0,429
Mean 3	0,7956	0,6006	0,8346	0,3744
Mean 4	0,7878	0,6864	0,7956	0,3978

SOD2

	group 1	group 2	group 3	group 4
Mean 1	0.9918	0.8178	0.4593	1.3439
Mean 2	0.9803	1.1998	0.5072	1.2233
Mean 3	0.98	0.9248	0.5485	1.2795
Mean 4	0.8799	0.8267	0.6499	1.3463

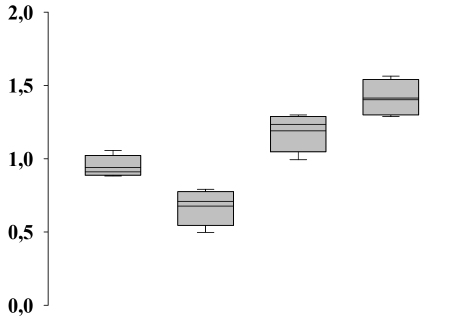

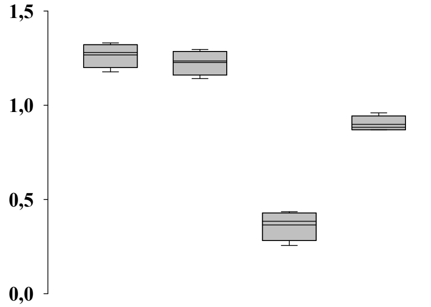

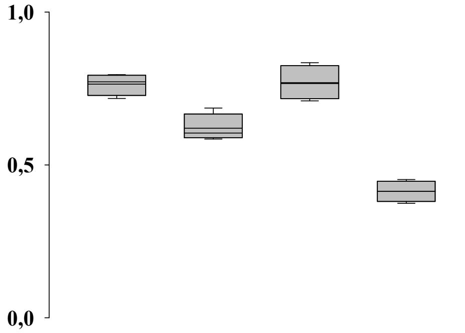

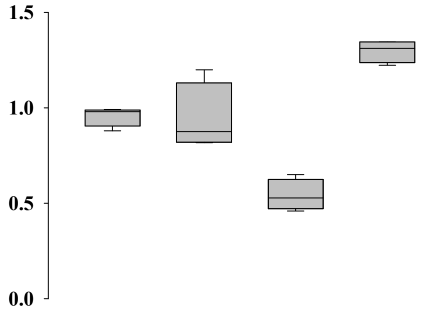

One Way Analysis of Variance All Pairwise Multiple Comparison Procedures (Student-Newman-Keuls Method) :

Comparison	Diff of Means	P<0.050
CNPase group 1 vs. CNPase group 3	0.251	Yes
CNPase group 1 vs. CNPase group 2	0.264	Yes
CNPase group 1 vs. CNPase group 4	0.474	Yes
CNPase group 4 vs. CNPase group 3	0.224	Yes

Comparison	Diff of Means	P<0.050
ANT group 1 vs. ANT group 3	0.902	Yes
ANT group 1 vs. ANT group 2	0.0402	No
ANT group 1 vs. ANT group 4	0.368	Yes
ANT group 4 vs. ANT group 3	0.534	Yes

Comparison	Diff of Means	P<0.050
CyP-D group 1 vs. CyP-D group 3	0.00487	No
CyP-D group 1 vs. CyP-D group 2	0.144	Yes
CyP-D group 1 vs. CyP-D group 4	0.351	Yes
CyP-D group 4 vs. CyP-D group 3	0.356	Yes

Comparison	Diff of Means	P<0.050
SOD2 group 1 vs. SOD2 group 3	0.417	Yes
SOD2 group 1 vs. SOD2 group 2	0.0157	No
SOD2 group 1 vs. SOD2 group 4	0.34	Yes
SOD2 group 4 vs. SOD2 group 3	0.757	Yes

To Fig 5.

	Activity I complex				ANT I complex				Cyc D I complex				NDUFB8 I complex			
	group 1	group 2	group 3	group 4	group 1	group 2	group 3	group 4	group 1	group 2	group 3	group 4	group 1	group 2	group 3	group 4
Mean 1	2,641	2,641	2,085	2,4464	0,8148	2,3086	0,1649	1,261	1,9306	1,0835	0,3349	0,985	0,817	0,9546	0,4988	0,8256
Mean 2	2,4742	2,7522	1,3344	2,502	0,8245	2,1243	0,1746	1,1349	1,9897	1,2017	0,3546	1,0244	0,8342	0,8342	0,5074	0,9546
Mean 3	2,98	2,8634	1,6402	2,4742	1,067	2,4929	0,3589	1,3095	2,167	1,1426	0,4925	0,8865	0,8944	0,9116	0,559	0,8514
Mean 4	2,9912	2,8356	1,6988	2,2796	1,1446	2,4153	0,2716	1,5229	2,0094	1,379	0,5713	0,8865	0,86	0,8514	0,5676	0,8084

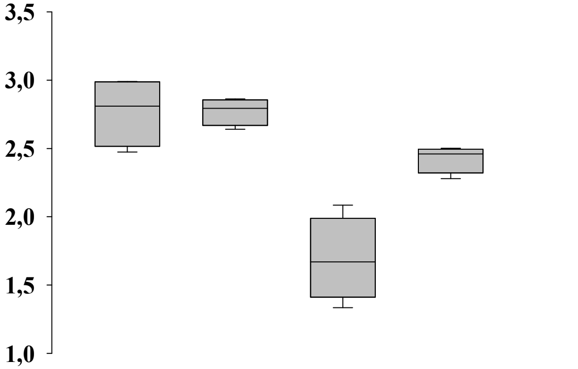

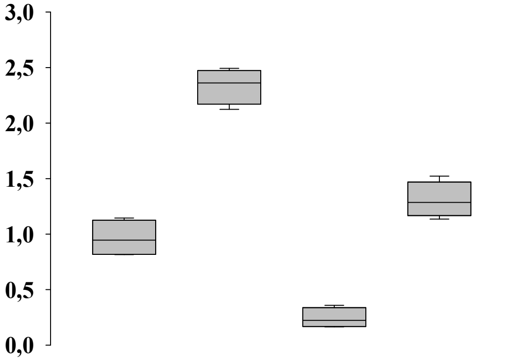

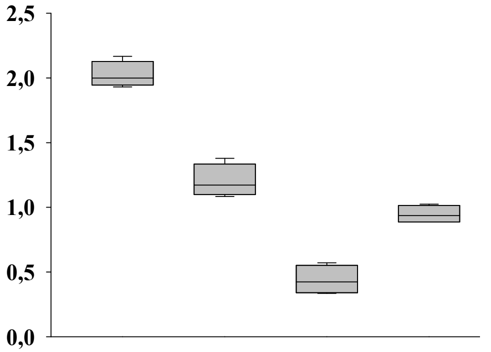

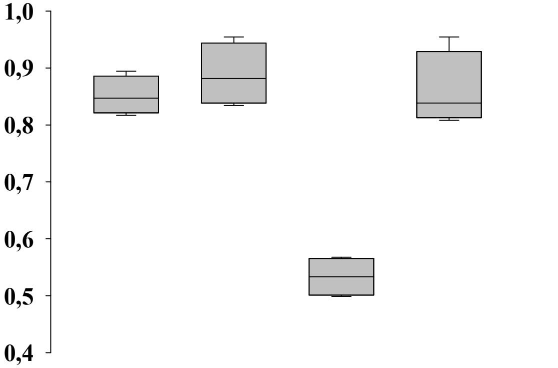

One Way Analysis of Variance All Pairwise Multiple Comparison Procedures (Student-Newman-Keuls Method) :

Comparison	Diff of Means	P<0.050
activity C I group 1 vs. activity C I group 3	1.082	Yes
activity C I group 1 vs. activity C I group 2	0.00145	No
activity C I group 1 vs. activity C I group 4	0.346	Yes
activity C I group 4 vs. activity C I group 3	0.736	Yes

Comparison	Diff of Means	P<0.050
ANT C I group 1 vs. ANT C I group 3	0.72	Yes
ANT C I group 1 vs. ANT C I group 2	1.373	Yes
ANT C I group 1 vs. ANT C I group 4	0.344	Yes
ANT C I group 4 vs. ANT C I group 3	1.065	Yes

Comparison	Diff of Means	P<0.050
CyP-D C I group 1 vs. CyP-D I group 3	1.586	Yes
CyP-D C I group 1 vs. CyP-D I group 2	0.822	Yes
CyP-D I group 1 vs. CyP-D I group 4	1.079	Yes
CyP-D I group 4 vs. CyP-D I group 3	0.507	Yes

Comparison	Diff of Means	P<0.050
NDUF C I group 1 vs. NDUF C I group 3	0.318	Yes
NDUF C I group 1 vs. NDUF C I group 2	0.0366	No
NDUF C I group 1 vs. NDUF C I group 4	0.814	No
NDUF C I group 4 vs. NDUF C I group 3	0.147	Yes

To Fig 6.

	Activity IV complex				COX IV				MTCO1 IV complex			
	group 1	group 2	group 3	group 4	group 1	group 2	group 3	group 4	group 1	group 2	group 3	group 4
Mean 1	0,66	0,6534	0,462	0,858	1,123	1,0781	0,73	0,8984	3,13	3,1116	1,252	2,0345
Mean 2	0,693	0,5808	0,4752	0,99	1,0332	1,0107	0,6513	0,8423	3,1926	3,0048	0,8451	2,4101
Mean 3	0,6402	0,6468	0,4488	0,9438	1,1118	0,9882	0,685	0,9209	3,0987	3,0987	1,0016	2,0658
Mean 4	0,7458	0,6072	0,5412	0,8316	1,1005	1,1455	0,8086	0,8759	3,2865	2,9109	1,3146	1,6276

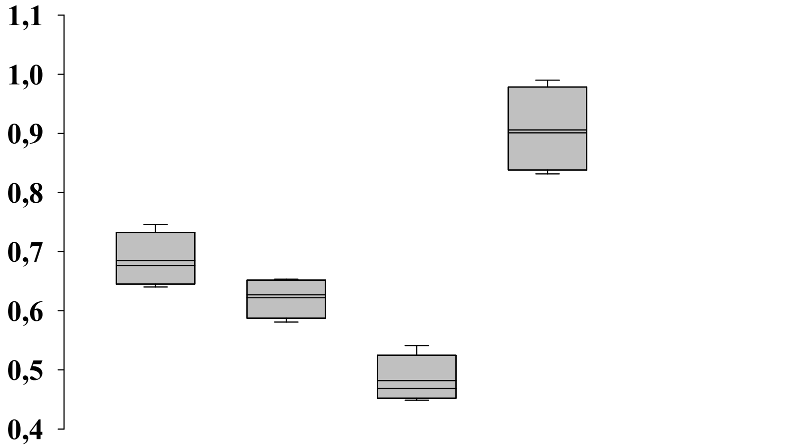

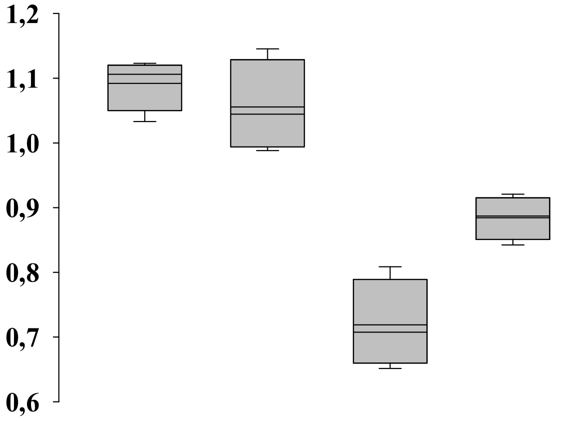

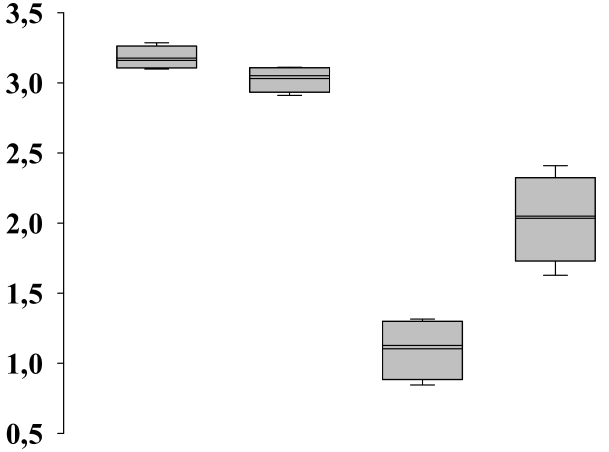

One Way Analysis of Variance All Pairwise Multiple Comparison Procedures (Student-Newman-Keuls Method) :

Comparison	Diff of Means	P<0.050
activity C IV group 1 vs. activity C IV group 3	0.203	Yes
activity C IV group 1 vs. activity C IV group 2	0.0627	No
activity C IV group 1 vs. activity C IV group 4	0.221	Yes
activity C IV group 4 vs. activity C IV group 3	0.424	Yes

Comparison	Diff of Means	P<0.050
COX C IV group 1 vs. COX C IV group 3	0.373	Yes
COX C IV group 1 vs. COX C IV group 2	0.0365	No
COX C IV group 1 vs. COX C IV group 4	0.11	Yes
COX C IV group 4 vs. COX C IV group 3	0.54	Yes

Comparison	Diff of Means	P<0.050
MTCO I C IV group 1 vs. MTCO I C IV group 3	2.074	Yes
MTCO I C IV group 1 vs. MTCO I C IV group 2	0.145	No
MTCO I C IV group 1 vs. MTCO I C IV group 4	1.142	Yes
MTCO I C IV group 4 vs. MTCO I C IV group 3	0.931	Yes

To Fig 7.

	Activity II complex				SDHB II complex			
	group 1	group 2	group 3	group 4	group 1	group 2	group 3	group 4
Mean 1	1,43	1,5344	1,144	1,4586	1,9602	1,98	1,5642	2,3364
Mean 2	1,5873	1,3871	1,1011	1,4872	1,8018	2,1582	1,5642	2,4552
Mean 3	1,4014	1,3728	1,0654	1,573	2,1384	2,075	1,6368	2,4156
Mean 4	1,4586	1,5064	1,2584	1,3013	1,9998	1,8303	1,4078	2,5233

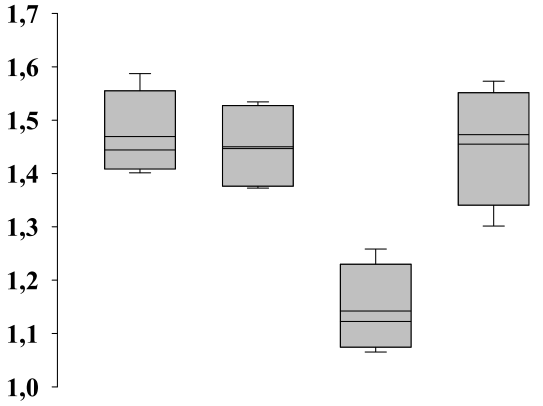

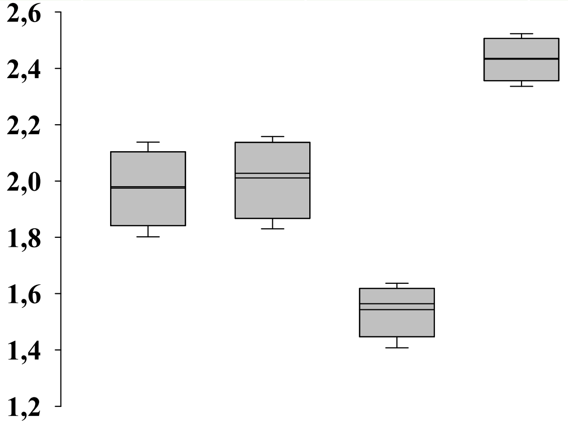

One Way Analysis of Variance All Pairwise Multiple Comparison Procedures (Student-Newman-Keuls Method) :

Comparison	Diff of Means	P<0.050
activity C II group 1 vs. activity C II group 3	0.327	Yes
activity C II group 1 vs. activity C II group 2	0.0192	No
activity C II group 1 vs. activity C II group 4	0.0143	No
activity C II group 4 vs. activity C II group 3	0.313	Yes

Comparison	Diff of Means	P<0.050
SDHB C II group 1 vs. SDHB C II group 3	0.432	Yes
SDHB C II group 1 vs. SDHB C II group 2	0.0358	No
SDHB C II group 1 vs. SDHB C II group 4	0.458	Yes
SDHB C II group 4 vs. SDHB C II group 3	0.889	Yes

To Fig 8.

	CNPase III complex				UQCRC2 III complex			
	group 1	group 2	group 3	group 4	group 1	group 2	group 3	group 4
Mean 1	1,1123	0,8456	1,0099	1,452	1,6666	1,4787	0,9694	1,3458
Mean 2	0,9922	0,9111	1,1235	1,4412	1,4951	1,5909	0,9365	1,2158
Mean 3	0,9233	0,7666	1,1211	1,44	1,7044	1,5933	1,0296	1,283
Mean 4	0,9455	0,7444	0,9444	1,5344	1,6594	1,5188	1,1682	1,2723

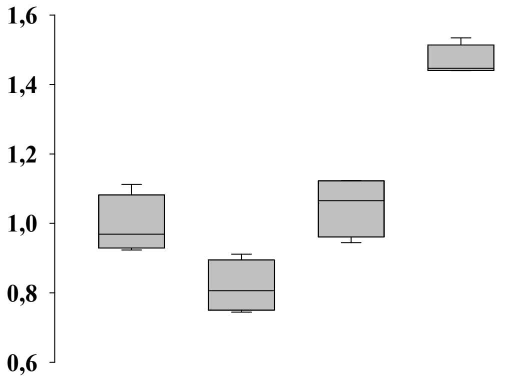

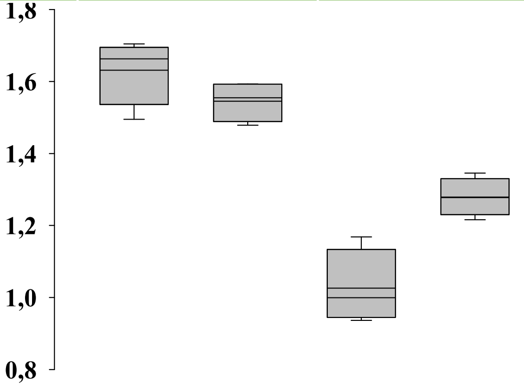

One Way Analysis of Variance All Pairwise Multiple Comparison Procedures (Student-Newman-Keuls Method) :

Comparison	Diff of Means	P<0.050
CNPase C III group 1 vs. CNPase C III group 3	0.0564	No
CNPase C III group 1 vs. CNPase C III group 2	0.176	Yes
CNPase C III group 1 vs. CNPase C III group 4	0.474	Yes
CNPase C III group 4 vs. CNPase C III group 3	0.417	Yes

Comparison	Diff of Means	P<0.050
UQCRC2 group 1 vs. UQCRC2 group 3	0.605	Yes
UQCRC2 group 1 vs. UQCRC2 group 2	0.086	No
UQCRC2 group 1 vs. UQCRC2 group 4	0.352	Yes
UQCRC2 group 4 vs. UQCRC2 group 3	0.253	Yes

To Fig 9.

	Activity V complex group 1	Activity V complex group 2	Activity V complex group 3	Activity V complex group 4
Mean 1	1,6758	1,6416	1,1115	1,71
Mean 2	1,6245	1,7955	1,2825	1,7784
Mean 3	1,7955	1,6758	1,368	1,539
Mean 4	1,7442	1,6929	1,3338	1,539

	ATP5G V complex group 1	ATP5G V complex group 2	ATP5G V complex group 3	ATP5G V complex group 4
Mean 1	1,078	3,003	0,715	2,398
Mean 2	1,144	3,586	0,792	2,068
Mean 3	1,309	3,652	0,847	2,068
Mean 4	1,155	3,63	0,968	2.2

	ATP5F1 V complex group 1	ATP5F1 V complex group 2	ATP5F1 V complex group 3	ATP5F1 V complex group 4
Mean 1	3,0586	3,0586	2,7465	3,7452
Mean 2	2,9337	3,0898	2,6529	3,6828
Mean 3	3,2146	3,2771	2,7465	3,87
Mean 4	3.1453	3.121	2.528	3.7764

	ATP5A V complex group 1	ATP5A V complex group 2	ATP5A V complex group 3	ATP5A V complex group 4
Mean 1	0,8026	0,913	0,332	1,2865
Mean 2	0,913	0,9379	0,3054	1,4168
Mean 3	0,8275	0,9296	0,3287	1,3571
Mean 4	0,8034	1,0375	0,4059	1,3853

	Cyc D V complex group 1	Cyc D V complex group 2	Cyc D V complex group 3	Cyc D V complex group 4
Mean 1	0,4998	0,4981	1,6044	0,4584
Mean 2	0,5197	0,5495	1,5929	0,4298
Mean 3	0,5776	0,6246	1,6445	0,4756
Mean 4	0,6406	0,6863	1,5414	0,4011

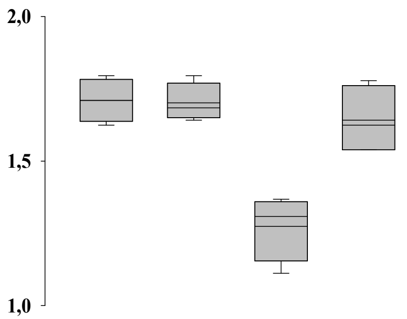

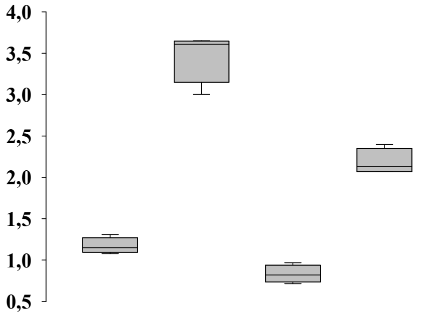

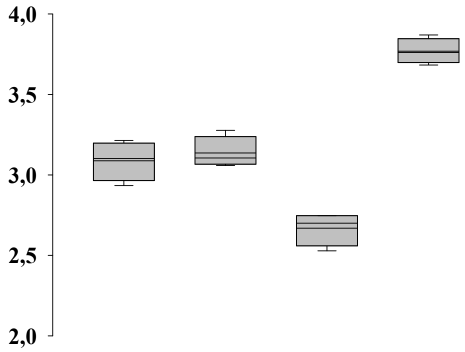

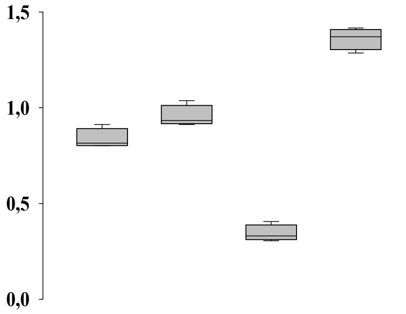

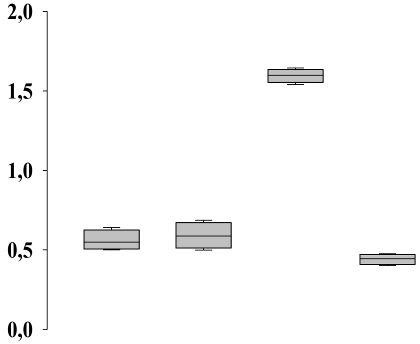

One Way Analysis of Variance All Pairwise Multiple Comparison Procedures (Student-Newman-Keuls Method) :

Comparison	Diff of Means	P<0.050
Activity C V group 1 vs. Activity C V group 3	0.436	Yes
Activity C V group 1 vs. Activity C V group 2	0.00855	No
Activity C V group 1 vs. Activity C V group 4	0.0684	No
Activity C V group 4 vs. Activity C V group 3	0.368	Yes

Comparison	Diff of Means	P<0.050
ATP5G C V group 1 vs. ATP5G C V group 3	1.626	Yes
ATP5G C V group 1 vs. ATP5G C V group 2	1.854	Yes
ATP5G C V group 1 vs. ATP5G C V group 4	0.9524	Yes
ATP5G C V group 4 vs. ATP5G C V group 3	0.998	Yes

Comparison	Diff of Means	P<0.050
ATP5F C V group 1 vs. ATP5F C V group 3	0.42	Yes
ATP5F C V group 1 vs. ATP5F C V group 2	0.0485	No
ATP5F C V group 1 vs. ATP5F C V group 4	0.681	Yes
ATP5F C V group 4 vs. ATP5F C V group 3	1.1	Yes

Comparison	Diff of Means	P<0.050
ATP5A C V group 1 vs. ATP5A C V group 3	0.494	Yes
ATP5A C V group 1 vs. ATP5A C V group 2	0.118	Yes
ATP5A C V group 1 vs. ATP5A C V group 4	0.525	Yes
ATP5A C V group 4 vs. ATP5A C V group 3	1.018	Yes

Comparison	Diff of Means	P<0.050
CyP-D C V group 1 vs. CyP-D C V group 3	1.036	Yes
CyP-D C V group 1 vs. CyP-D C V group 2	0.0302	No
CyP-D C V group 1 vs. CyP-D C V group 4	0.118	Yes
CyP-D C V group 4 vs. CyP-D C V group 3	1.115	Yes
